# National Trends in Racial and Ethnic Disparities in Use of Recommended Therapies in Adults with Atherosclerotic Cardiovascular Disease, 1999-2020

**DOI:** 10.1001/jamanetworkopen.2023.45964

**Published:** 2023-12-01

**Authors:** Yuan Lu, Yuntian Liu, Lovedeep Singh Dhingra, César Caraballo, Shiwani Mahajan, Daisy Massey, Erica S. Spatz, Richa Sharma, Fatima Rodriguez, Karol E. Watson, Frederick A. Masoudi, Harlan M. Krumholz

**Affiliations:** 1 Center for Outcomes Research and Evaluation, Yale New Haven Hospital, New Haven, Connecticut; 2 Section of Cardiovascular Medicine, Department of Internal Medicine, Yale School of Medicine, New Haven, Connecticut; 3 Department of Neurology, Yale School of Public Health, New Haven, Connecticut; 4 Division of Cardiovascular Medicine, School of Medicine, Stanford University, Stanford, California; 5 David Geffen School of Medicine, University of California, Los Angeles; 6 Research and Analytics, Ascension, St Louis, Missouri; 7 Department of Health Policy and Management, Yale School of Public Health, New Haven, Connecticut

## Abstract

**Importance:**

Despite efforts to improve the quality of care for patients with atherosclerotic cardiovascular disease (ASCVD), it is unclear whether the US has made progress in reducing racial and ethnic differences in utilization of guideline-recommended therapies for secondary prevention.

**Objective:**

To evaluate 21-year trends in racial and ethnic differences in utilization of guideline-recommended pharmacological medications and lifestyle modifications among US adults with ASCVD.

**Design, Setting, and Participants:**

This cross-sectional study includes data from the National Health and Nutrition Examination Survey between 1999 and 2020. Eligible participants were adults aged 18 years or older with a history of ASCVD. Data were analyzed between March 2022 and May 2023.

**Exposure:**

Self-reported race and ethnicity.

**Main Outcome and Measures:**

Rates and racial and ethnic differences in the use of guideline-recommended pharmacological medications and lifestyle modifications.

**Results:**

The study included 5218 adults with a history of ASCVD (mean [SD] age, 65.5 [13.2] years, 2148 women [weighted average, 44.2%]), among whom 1170 (11.6%) were Black, 930 (7.7%) were Hispanic or Latino, and 3118 (80.7%) were White in the weighted sample. Between 1999 and 2020, there was a significant increase in total cholesterol control and statin use in all racial and ethnic subgroups, and in angiotensin-converting enzyme inhibitor (ACEI) and angiotensin receptor blocker (ARB) utilization in non-Hispanic White individuals and Hispanic and Latino individuals (Hispanic and Latino individuals: 17.12 percentage points; 95% CI, 0.37-37.88 percentage points; *P* = .046; non-Hispanic White individuals: 12.14 percentage points; 95% CI, 6.08-18.20 percentage points; *P* < .001), as well as smoking cessation within the Hispanic and Latino population (−27.13 percentage points; 95% CI, −43.14 to −11.12 percentage points; *P* = .002). During the same period, the difference in smoking cessation between Hispanic and Latino individuals and White individuals was reduced (−24.85 percentage points; 95% CI, −38.19 to −11.51 percentage points; *P* < .001), but racial and ethnic differences for other metrics did not change significantly. Notably, substantial gaps persisted between current care and optimal care throughout the 2 decades of data analyzed. In the period of 2017 to 2020, optimal regimens were observed in 47.4% (95% CI, 39.3%-55.4%), 48.7% (95% CI, 36.7%-60.6%), and 53.0% (95% CI, 45.6%-60.4%) of Black, Hispanic and Latino, and White individuals, respectively.

**Conclusions and Relevance:**

In this cross-sectional study of US adults with ASCVD, significant disparities persisted between current care and optimal care, surpassing any differences observed among demographic groups. These findings highlight the critical need for sustained efforts to bridge these gaps and achieve better outcomes for all patients, regardless of their racial and ethnic backgrounds.

## Key Points

**Question:**

How have racial and ethnic disparities in the utilization of guideline-recommended therapies for secondary prevention among US adults with atherosclerotic cardiovascular disease (ASCVD) changed over the past 2 decades?

**Findings:**

In this cross-sectional study of 5218 US adults with ASCVD, we observed significant improvements in cholesterol control, statin use, and angiotensin-converting enzyme inhibitor and angiotensin receptor blocker use among all racial and ethnic subgroups, as well as smoking cessation among Hispanics and Latino individuals. However, a substantial gap persisted between current care and optimal care, with only 50% of patients overall adopting optimal prevention strategies across racial and ethnic subgroups.

**Meaning:**

These results suggest that, despite efforts to enhance the quality of care for ASCVD patients, substantial disparities persist between current care and optimal care, underscoring the urgent need for ongoing initiatives to bridge these gaps and improve outcomes for all patients, regardless of their backgrounds.

## Teaser

This cross-sectional study of adults with atherosclerotic cardiovascular disease (ASCVD) compares adoption of recommended prevention guidelines and optimal care practices across racial and ethnic categories.

## Introduction

Individuals with atherosclerotic cardiovascular disease (ASCVD) are at high risk for subsequent cardiovascular events and mortality.^1^ Evidence-based secondary prevention guidelines recommend the use of lifestyle modifications and pharmacological medications to reduce risk factors such as hypertension, dyslipidemia, and diabetes.^2^ However, previous studies have shown substantial racial and ethnic disparities in adherence to these guidelines,^3,4^ with non-Hispanic Black patients and Hispanic and Latino patients being less likely to achieve cardiovascular risk factor control goals compared with non-Hispanic White patients.^5,6^ Given the national efforts to eliminate health disparities^7,8,9^ and the American Heart Association’s goal to champion health equity and advance cardiovascular health for all,^10^ it is crucial to assess how much progress has been made in eliminating disparities in guideline-recommended therapies for secondary prevention among patients with ASCVD.

Limited national-level data exist on racial and ethnic disparities in adherence to secondary prevention guidelines, with available data only extending through 2012.^11,12^ Since that time, new evidence-based guidelines for hypertension,^13,14^ hypercholesterolemia,^15^ and secondary prevention of coronary artery disease and stroke have been introduced.^2,16,17^ Despite the introduction of these new guidelines, no recent comprehensive studies have evaluated the utilization of guideline-recommended therapies by race and ethnicity. Previous studies, while informative, have focused on only a small number of medications, such as statins, and have not systematically quantified racial and ethnic disparities in the use of a broad range of therapies for secondary prevention.^12,18^

Therefore, this study aims to estimate 21-year trends in the utilization of guideline-recommended therapies for secondary prevention among US adults with ASCVD and to compare these trends by race and ethnicity using the National Health and Nutrition Examination Surveys (NHANES) from 1999 to 2020. This study addressed gaps in previous research by systematically quantifying racial and ethnic differences in the use of a wide range of therapies for secondary prevention, including pharmacological medications and lifestyle modifications.

## Methods

### Study Design and Population

We analyzed data from the National Health and Nutrition Examination Survey (NHANES) for the years 1999 to March 2020, which enrolled a total of 63 041 adults aged 18 years and older.^19^ Detailed data collection in NHANES and definitions of other covariates are reported in eTable 1 and eMethods in Supplement 1. This study received an exemption from review by the institutional review board at Yale University because NHANES data are publicly available and deidentified. In accordance with best practices in reporting, this paper adheres to the Strengthening the Reporting of Observational Studies in Epidemiology (STROBE) reporting guideline.

Participants’ self-reported history of coronary heart disease, heart attack, or stroke was used to define a history of ASCVD. We categorized participants into 3 mutually exclusive racial and ethnic subgroups based on their self-reported race and ethnicity: non-Hispanic Black (hereafter Black), Hispanic and Latino, and non-Hispanic White (hereafter White). To ensure adequate sample sizes, individuals who identified as Asian, Alaskan Native or American Indian, or other were excluded from the analysis.

### Treatment and Control of Cardiovascular Risk Factors

To determine treatment rates for cardiovascular risk factors, we assessed participants’ self-reported use of pharmacotherapy or examined prescription medication bottles during the interview. Following previous guidelines for ASCVD prevention, we defined target risk factor levels for control as a systolic blood pressure below 140 mm Hg and a diastolic blood pressure below 90 mm Hg for hypertension, total cholesterol below 200 mg/dL (to convert to millimoles per liter, multiply by 0.0259) for hypercholesterolemia, and hemoglobin A_1c_ below 7% (to convert to proportion of total hemoglobin, multiply by 0.01) for diabetes. We used total cholesterol to calculate hypercholesterolemia control rates. We did not use low-density lipoprotein cholesterol in the calculation because those data were only available in a subset of individuals.

### Use of Individual Medication

Secondary prevention guidelines recommend the use of aspirin and statin in all patients with ASCVD without contraindications (see eTable 2 in Supplement 1).^2^ Therefore, we considered all adults with ASCVD to have an indication for aspirin and statin use. Secondary prevention guidelines also recommend the use of angiotensin-converting enzyme inhibitors (ACEI) or angiotensin receptor blockers (ARB) in patients with ASCVD who have left ventricular ejection fraction of 40% or below and in those with hypertension, diabetes, or chronic kidney disease. As information on ejection fraction was not available in NHANES, we considered adults with ASCVD and at least 1 of hypertension, diabetes, or chronic kidney disease to have an indication for ACEI or ARB. Treatment rates for statin, aspirin, and ACEI or ARB were calculated among those with an indication for treatment. Optimal regimen use was defined as being treated with aspirin, a cholesterol-lowering medication, and a blood pressure–lowering medication among eligible participants. As NHANES only conducted a questionnaire on preventive aspirin use after 2011, the use of aspirin and optimal regimen were reported from 2011 to 2020.

### Statistical Analysis

We conducted all analyses using appropriate methods for structured survey data, incorporating strata and weights to produce nationally representative estimates.^19^ We first described the sociodemographic and clinical characteristics of adults with ASCVD by race and ethnicity. We reported mean and standard deviation for continuous variables and percentage and confidence interval for categorical variables. Given that these percentages were estimated from NHANES using sampling weights to achieve national representativeness, they inherently carry a degree of associated uncertainty. Next, we estimated the age-adjusted annual use rates of guideline-recommended therapies and risk factor control rates by racial and ethnic subgroups using multivariable linear regression models. For each racial and ethnic subgroup and outcome, we estimated a separate model that included standardized age and an indicator for each survey year as independent variables. The coefficients for each year represented the age-adjusted annual rates for the designated outcome.^20^ To estimate the trend for each outcome, we used a weighted linear regression, using the reciprocal of the annual rate’s standard error as weights. We reported the results in 5 time intervals (1999-2004, 2005-2008, 2009-2012, 2013-2016, and 2017-2020) to achieve sufficient sample size. Finally, we calculated the racial and ethnic differences in use rates of guideline-recommended therapies and risk factor control rates, using White individuals as the reference group, and we assessed how racial and ethnic differences changed over time. We also assessed the trends in social determinants of health, encompassing education, family income, insurance status, employment status and marital status.

We considered 2-sided *P* values < .05 to be statistically significant. All analyses were performed using R version 4.0 (R Project for Statistical Computing).

## Results

Out of 63 041 adults in NHANES (1999-2020), we focused on 5218 adults with ASCVD after excluding pregnant women, certain racial groups, and those without ASCVD history (eFigure 1 in Supplement 1). This group had a mean (SD) age of 65.5 (13.2) years, with 2148 women (weighted average, 44.2%) (Table 1). Black individuals constituted 11.6% (95% CI, 10.2%-13.1%) of the weighted sample, Hispanic and Latino individuals accounted for 7.7% (6.4%-8.9%), and White individuals comprised 80.7% (78.7%-82.7%). Black individuals and Hispanic and Latino individuals were younger, less educated, more often uninsured, physically inactive, and had lower income and higher diabetes and hypertension rates compared with White individuals (Table 1; eTable 3 in Supplement 1).

**Table 1.** Sociodemographic Characteristics of Adults Reporting Prior Atherosclerotic Cardiovascular Disease in 1999-2020

Characteristics	Individuals, No. (estimated %) [95% CI] (N = 5218)		
	Non-Hispanic White	Non-Hispanic Black	Hispanic or Latino
Total sample	3118 (80.7) [78.7-82.7]	1170 (11.6) [10.2-13.1]	930 (7.7) [6.4-8.9]
Mean (SD) age, y	66.7 (12.8)	60.8 (13.4)	59.7 (14.5)
Sex			
Women	1208 (42.9) [40.4-45.4]	541 (53.1) [49.1-57.1]	399 (45.0) [40.2-49.9]
Men	1910 (57.1) [54.6-59.6]	629 (46.9) [42.9-50.9]	531 (55.0) [50.1-59.8]
Education level			
More than high school	1363 (47.5) [44.9-50.2]	395 (35.8) [32.3-39.4]	208 (26.0) [21.2-30.9]
High school	889 (30.4) [28.3-32.5]	291 (24.6) [21.3-27.9]	140 (19.9) [15.3-24.4]
Less than high school	861 (22.1) [19.8-24.3]	475 (39.6) [35.5-43.7]	577 (54.1) [48.3-59.9]
Family income			
High or middle income	1413 (60.3) [57.3-63.4]	400 (38.4) [33.9-42.8]	225 (28.2) [23-33.4]
Low income	1437 (39.7) [36.6-42.7]	641 (61.6) [57.2-66.1]	576 (71.8) [66.6-77]
Insurance status			
Insured	2947 (94.3) [93.1-95.5]	1063 (89.4) [87.4-91.4]	778 (79.9) [75.3-84.5]
Uninsured	155 (5.7) [4.5-6.9]	96 (10.6) [8.6-12.6]	144 (20.1) [15.5-24.7]
Marital status			
Married or living with partner	1783 (62.6) [60.1-65.1]	466 (39.8) [35.9-43.7]	542 (58.2) [53.2-63.3]
Unmarried	1314 (37.4) [34.9-39.9]	686 (60.2) [56.3-64.1]	373 (41.8) [36.7-46.8]
Employment status			
Not in labor force	2495 (71.0) [68.6-73.4]	902 (74.8) [71.1-78.6]	698 (69.9) [64.9-74.9]
Unemployed	36 (1.5) [0.9-2.1]	35 (3.5) [2-4.9]	24 (3.5) [1.3-5.8]
With a job or working	563 (27.5) [25.1-29.9]	218 (21.7) [18.4-25.1]	183 (26.5) [21.9-31.2]

### Trends in Racial and Ethnic Differences in Treatment and Control of Cholesterol

**Figure 1.**
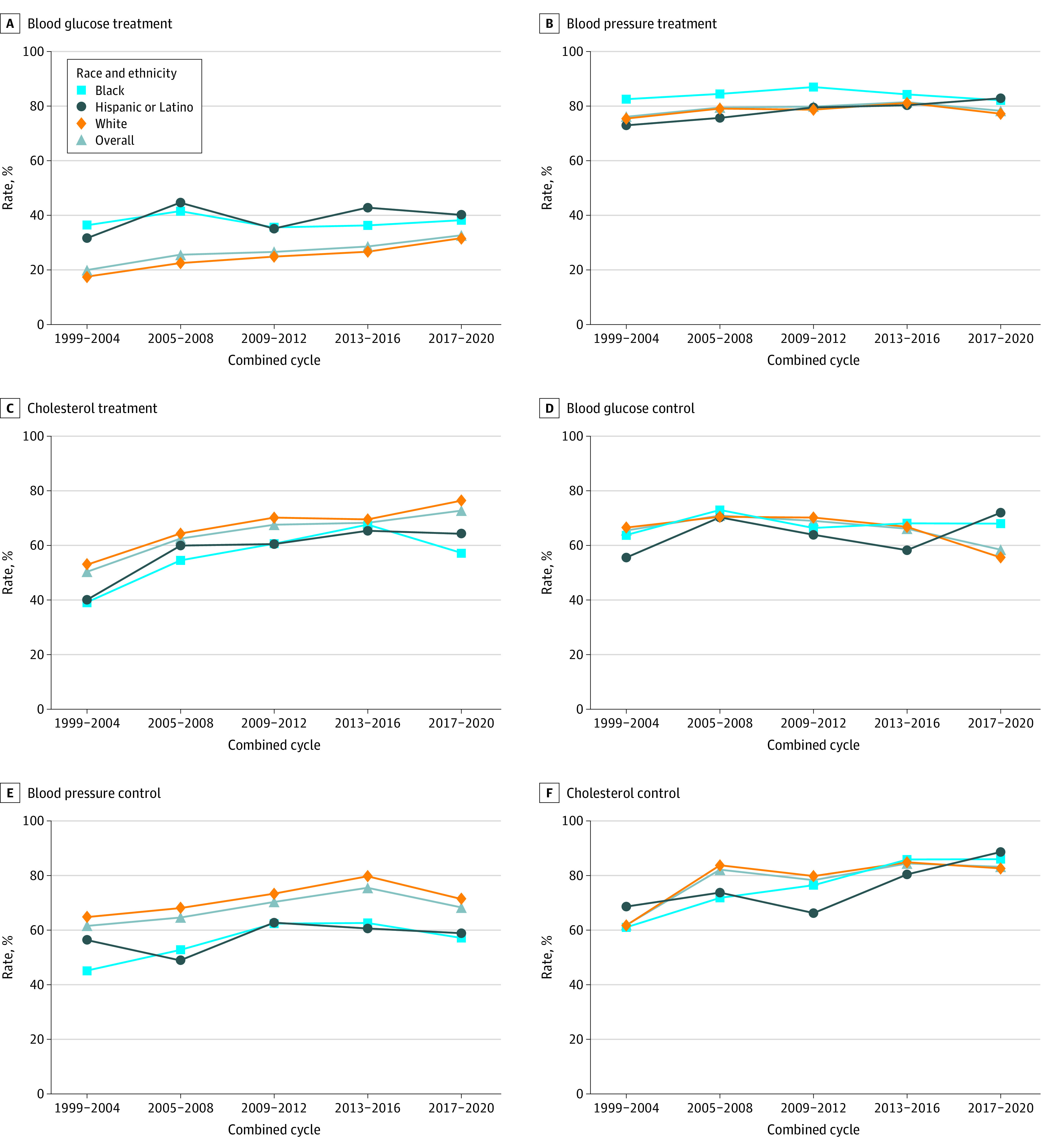
Trends in Treatment and Control of Cholesterol, Blood Pressure, and Blood Glucose Among Adults With Atherosclerotic Cardiovascular Disease by Race and Ethnicity, 1999-2020

From 1999 to 2004, the age-adjusted use rate of lipid-lowering medications was 39.3% (95% CI, 32.1%-46.6%) in Black individuals, 40.1% (95% CI, 20.8%-59.3%) in Hispanic and Latino individuals, and 52.9% (95% CI, 49.4%-56.5%) in White individuals, respectively (Figure 1; eTable 4 in Supplement 1). A significant increase in the use rate of lipid-lowering medications and the control rate of total cholesterol among those treated was observed among all racial and ethnic subgroups between 1999 and 2020 (Black, 18.31 percentage points; 95% CI, 7.42 to 29.19 percentage points; Hispanic or Latino, 26.02 percentage points; 95% CI, 6.73 to 45.31 percentage points; White, 23.50 percentage points; 95% CI, 16.56 to 30.45 percentage points) (Table 2). However, no significant changes were observed in the difference in treatment and control rates of cholesterol between White and Black individuals and between White individuals and Hispanic and Latino individuals during the study period. Compared with White individuals, the use rate of lipid-lowering medications between 2017 and 2020 remained significantly lower among Black individuals (−24.07 percentage points; 95% CI, −29.12 to −14.52 percentage points; *P* < .001) and Hispanic and Latino individuals (−17.56 percentage points; 95% CI, −29.12 to −5.99 percentage points; *P* = .005) (Table 2).

**Table 2.** Change in Treatment and Control of Cholesterol, Blood Pressure, and Blood Glucose Among Adults With Atherosclerotic Cardiovascular Disease by Race and Ethnicity, 1999-2020

Characteristic	Non-Hispanic Black individuals, percentage points (95% CI)	*P* value	Hispanic and Latino individuals, percentage points (95% CI)	*P* value	Non-Hispanic White individuals, percentage points (95% CI)	*P* value
Use of lipid-lowering medication						
Absolute change in prevalence, 1999-2020	18.31 (7.42 to 29.19)	.001	26.02 (6.73 to 45.31)	.010	23.50 (16.56 to 30.45)	<.001
Difference with White, 1999-2004	−19.10 (−27.59 to −10.61)	<.001	−20.07 (−32.56 to −7.58)	.002	NA	NA
Difference with White, 2017-2020	−24.07 (−29.12 to −14.52)	<.001	−17.56 (−29.12 to −5.99)	.005	NA	NA
Absolute change in difference with White, 1999-2020	−5.20 (−17.84 to 7.44)	.76	2.52 (−14.15 to 19.18)	.42	NA	NA
Control of total cholesterol among treated individuals						
Absolute change in prevalence, 1999-2020	25.89 (9.71 to 42.08)	.003	20.70 (0 to 42.36)	.05	21.50 (13.72 to 29.28)	<.001
Difference with White, 1999-2004	−1.80 (−15.27 to 11.68)	.79	5.67 (−9.80 to 21.13)	.74	NA	NA
Difference with White, 2017-2020	1.32 (−9.38 to 12.03)	.80	4.57 (−7.07 to 16.21)	.43	NA	NA
Absolute change in difference with White, 1999-2020	4.39 (−12.01 to 20.79)	.60	−0.81 (−18.04 to 16.43)	.93	NA	NA
Use of blood pressure–lowering medication						
Absolute change in prevalence, 1999-2020	−0.20 (−11.37 to 10.98)	.97	11.78 (−4.84 to 28.40)	.16	1.87 (−4.13 to 7.88)	.54
Difference with White, 1999-2004	1.75 (−5.64 to 9.14)	.64	−10.18 (−20.36 to −0.01)	.05	NA	NA
Difference with White, 2017-2020	−0.32 (−10.40 to 9.76)	.95	−0.28 (−11.53 to 10.97)	.96	NA	NA
Absolute change in difference with White, 1999-2020	−2.07 (−14.24 to 10.10)	.74	9.91 (−4.91 to 24.72)	.19	NA	NA
Control of blood pressure among treated individuals						
Absolute change in prevalence, 1999-2020	11.85 (−3.61 to 27.31)	.13	2.13 (−20.83 to 25.09)	.85	6.97 (−0.77 to 14.71)	.08
Difference with White, 1999-2004	−16.35 (−26.82 to −5.88)	.003	−3.70 (−18.45 to 11.06)	.62	NA	NA
Difference with White, 2017-2020	−12.57 (−23.02 to −2.11)	.02	−9.54 (−24.00 to 4.91)	.19	NA	NA
Absolute change in difference with White, 1999-2020	4.88 (−10.96 to 20.72)	.54	−4.84 (−27.30 to 17.62)	.67	NA	NA
Use of blood glucose–lowering medication						
Absolute change in prevalence, 1999-2020	2.04 (−9.78 to 13.86)	.73	9.88 (−6.24 to 26.00)	.22	14.12 (8.48 to 19.75)	<.001
Difference with White, 1999-2004	14.96 (6.37 to 23.54)	.001	9.25 (0.01 to 18.48)	.05	NA	NA
Difference with White, 2017-2020	2.88 (−8.36 to 14.12)	.60	5.01 (−5.86 to 15.89)	.35	NA	NA
Absolute change in difference with White, 1999-2020	−12.08 (−25.86 to 1.71)	.09	−4.24 (−18.16 to 9.69)	.55	NA	NA
Control of blood glucose among treated individuals						
Absolute change in prevalence, 1999-2020	4.06 (−25.93 to 34.05)	.77	16.79 (−30.75 to 64.33)	.44	−11.20 (−28.57 to 6.17)	.199
Difference with White, 1999-2004	−3.15 (−19.26 to 12.96)	.69	−12.15 (−40.50 to 16.31)	.39	NA	NA
Difference with White, 2017-2020	12.12 (−4.70 to 28.93)	.15	15.84 (−7.25 to 38.93)	.17	NA	NA
Absolute change in difference with White, 1999-2020	15.26 (−7.40 to 37.93)	.18	27.99 (−7.78 to 63.76)	.12	NA	NA

Abbreviation: NA, not applicable.

### Trends in Racial and Ethnic Differences in Treatment and Control of Blood Pressure

From 1999 to 2004, the age-adjusted use rate of blood pressure–lowering medications was 82.5% (95% CI, 76.5%-88.6%) in Black individuals, 72.6% (95% CI, 57.4%-87.8%) in Hispanic and Latino individuals, and 75.4% (72.3%-78.6%) in White individuals, respectively (Figure 1; eTable 5 in Supplement 1). The treatment rates for blood pressure increased for Hispanic and Latino individuals and White individuals from 1999 to 2020, but these increases were not statistically significant (Table 2). For Black individuals, treatment rates increased until the 2009-2012 interval, followed by a decline between 2017 and 2020. Blood pressure control rates among those treated increased for all racial and ethnic subgroups between the 1999-2004 and 2013-2016 intervals but declined between 2017 and 2020. No significant changes were observed in the treatment and control rates of blood pressure between Black and White individuals or between Hispanic and Latino individuals and White individuals from 1999 to 2020 (Table 2). From 2017 to 2020, the control rate of blood pressure was lower among treated Black individuals by 12.57 percentage points (95% CI, −23.02 to −2.11 percentage points; *P* = .02) and Hispanic and Latino individuals by 9.54 percentage points (95% CI, −24.00 to 4.91 percentage points; *P* = .19) as compared with that of treated White individuals (71.4%; 95% CI, 65.0%-77.8%).

### Trends in Racial and Ethnic Differences in Treatment and Control of Blood Glucose

From 1999 to 2004, the age-adjusted use rate of glucose-lowering medications was 36.3% (95% CI, 27.8%-44.8%) in Black individuals, 31.3% (95% CI, 17.9%-44.8%) in Hispanic and Latino individuals, and 17.5% (95% CI, 14.2%-20.7%) in White individuals, respectively (Figure 1; eTable 6 in Supplement 1). From 1999 to 2020, treatment rates increased significantly for White individuals (14.12 percentage points; 95% CI, 8.48 to 19.75 percentage points; *P* < .001) but remained stagnant for Black individuals (Table 2). For Hispanic and Latino individuals, the treatment rate first increased from 31.3% (95% CI, 17.9%-44.8%) in the 1999-2004 interval to 44.3% (95% CI, 34.5%-54.2%) in 2005-2008, followed by a decline in 2009-2012, reaching 40.0% (95% CI, 29.5%-50.5%) in 2017-2020. In the same period, the gap in treatment rates between White and Black individuals narrowed due to increased usage among White individuals, although these results were not significant. Blood glucose control rates among those treated improved for Hispanic and Latino individuals but not significantly (Figure 1 and Table 2). From 2017 to 2020, the use rate of blood glucose–lowering medications among Black individuals and Hispanic and Latino individuals was not statistically different from that among White individuals, with rates of 31.6% (95% CI, 27.0%-36.1%), 28.8% (23.7%-34.0%), and 34.4% (30.6%-38.2%), respectively.

### Trends in Racial and Ethnic Differences in Individual Medication Use and Modifiable Risk Factors

**Figure 2.**
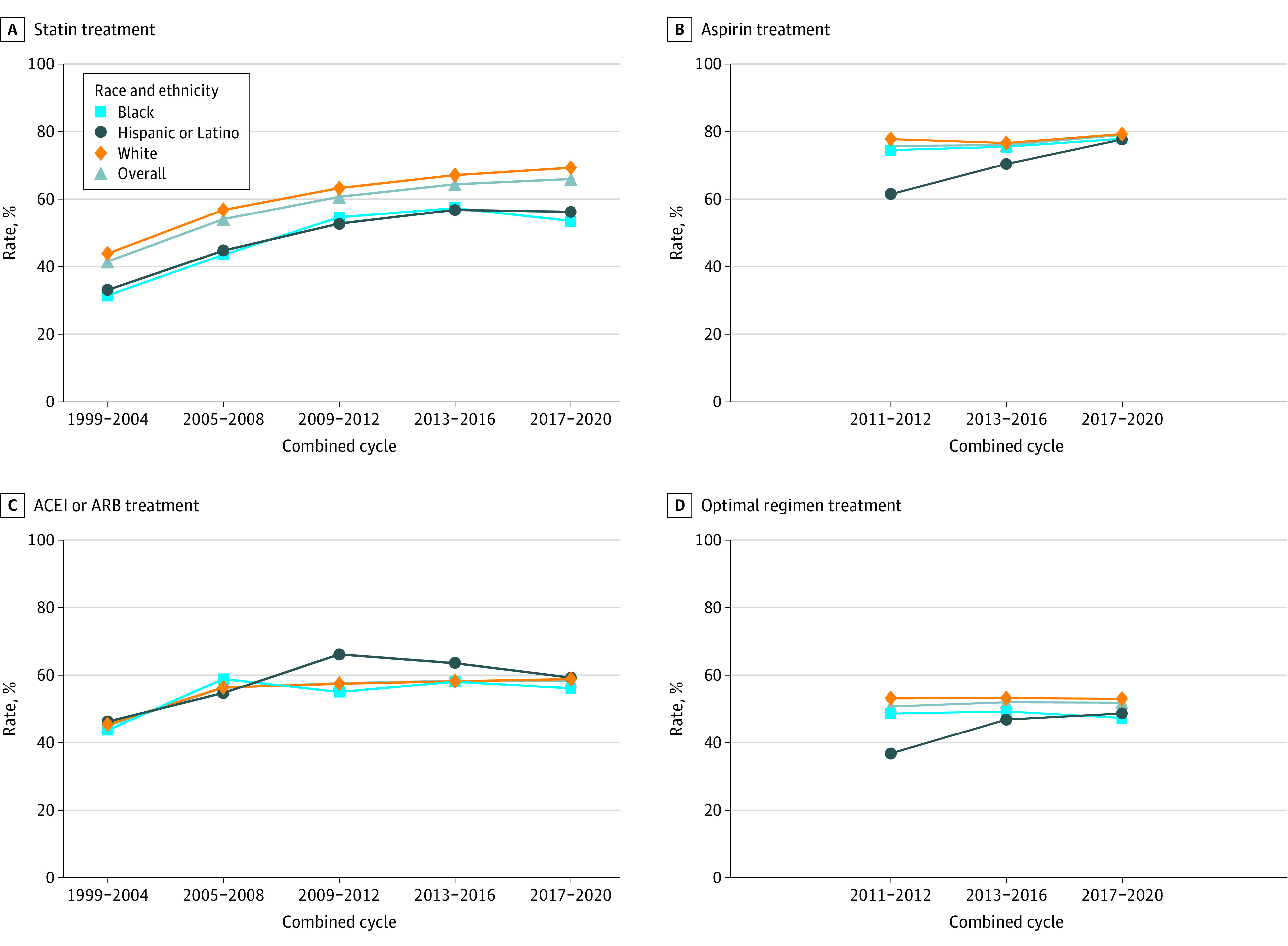
Trends in Use of Recommended Therapies Among Adults With Atherosclerotic Cardiovascular Disease by Race and Ethnicity, 1999-2020 As NHANES only conducted questionnaire for preventive aspirin use after year 2011, use of aspirin and optimal regimen were reported from 2011 to 2020.

Figure 2 illustrates trends in the use of statins, aspirin, and ACEI or ARB medications, as well as optimal regimen for individuals with ASCVD. Statin and ACEI or ARB use increased significantly from 1999 to 2020 for Hispanic or Latino (statin, 24.79 percentage points; 95% CI, 8.45 to 41.13; *P* = .004; ACEI or ARB, 17.12 percentage points; 95% CI, 0.37 to 33.88 percentage points; *P* = .046) and White individuals (statin, 25.45 percentage points; 95% CI, 18.62 to 32.28 percentage points; *P* < .001; ACEI or ARB, 12.14 percentage points; 95% CI, 6.08 to 18.20 percentage points; *P* < .001); while statin use increased among Black individuals (22.26 percentage points; 95% CI, 11.29 to 33.23 percentage points; *P* < .001), results for ACEI or ARB use were not significant (11.52 percentage points; 95% CI, −0.87 to 23.91 percentage points; *P* = .07) (Table 3). However, disparities persisted, with lower usage among Black individuals and Hispanic and Latino individuals from 2017 to 2020. The use rate of statins was lower by 20.62 percentage points (95% CI, −29.80 to −11.44 percentage points; *P* < .001) for Black individuals and 17.83 percentage points (95% CI, −28.02 to −7.63 percentage points; *P* < .001) for Hispanic and Latino individuals, respectively, compared with White individuals (69.2%; 95% CI, 64.1%-74.4%) (Table 3).

**Table 3.** Change in Pharmacological Medications, and Modifiable Lifestyle Risk Factors Among Adults With Atherosclerotic Cardiovascular Disease by Race and Ethnicity, 1999-2020

Characteristic	Non-Hispanic Black individuals, percentage points (95% CI)	*P* value	Hispanic and Latino individuals, percentage points (95% CI)	*P* value	Non-Hispanic White individuals, percentage points (95% CI)	*P* value
**Individual pharmacological medications**						
Use of statin						
Absolute change in prevalence, 1999-2020	22.26 (11.29 to 33.23)	<.001	24.79 (8.45 to 41.13)	.004	25.45 (18.62 to 32.28)	<.001
Difference with White, 1999-2004	−17.43 (−26.37 to −8.49)	<.001	−17.17 (−27.83 to −6.50)	.002	NA	NA
Difference with White, 2017-2020	−20.62 (−29.80 to −11.44)	<.001	−17.83 (−28.02 to −7.63)	.001	NA	NA
Absolute change in difference with White, 1999-2020	−3.19 (−15.71 to 9.33)	.61	−0.66 (−15.10 to 13.78)	.93	NA	NA
Use of ACEI or ARB						
Absolute change in prevalence, 1999-2020	11.52 (−0.87 to 23.91)	.07	17.12 (0.37 to 33.88)	.046	12.14 (6.08 to 18.20)	<.001
Difference with White, 1999-2004	−0.37 (−6.92 to 6.19)	.91	−3.17 (−13.61 to 7.26)	.54	NA	NA
Difference with White, 2017-2020	−0.98 (−11.94 to 9.98)	.86	1.81 (−11.46 to 15.09)	.78	NA	NA
Absolute change in difference with White, 1999-2020	−0.61 (−13.03 to 11.80)	.92	4.99 (−11.48 to 21.45)	.55	NA	NA
Use of aspirin						
Absolute change in prevalence, 2011-2020	3.25 (−10.75 to 17.26)	.64	15.65 (0.91 to 32.21)	.05	1.20 (−7.30 to 9.71)	.78
Difference with White, 2011-2012	−4.92 (−18.08 to 8.24)	.43	−18.20 (−29.64 to 6.76)	.004	NA	NA
Difference with White, 2017-2020	−2.87 (−13.15 to 7.41)	.57	−3.76 (−18.30 to 10.79)	.60	NA	NA
Absolute change in difference with White, 2011-2020	2.05 (−13.91 to 18.01)	.80	14.45 (−3.43 to 32.32)	.11	NA	NA
Use of optimal regimen						
Absolute change in prevalence, 2011-2020	−1.51 (−16.75 to 13.73)	.84	10.93 (−7.85 to 29.71)	.24	−0.46 (−10.84 to 9.93)	.93
Difference with White, 2011-2012	−7.13 (−22.67 to 8.41)	.34	−18.79 (−35.70 to −18.74)	.03	NA	NA
Difference with White, 2017-2020	−8.19 (−13.19 to 28.17)	.14	−7.41 (−22.51 to 7.70)	.32	NA	NA
Absolute change in difference with White, 2011-2020	−1.05 (−19.22 to 17.11)	.91	11.38 (−10.35 to 33.12)	.30	NA	NA
**Modifiable risk factors**						
Insufficient physical activity						
Absolute change in prevalence, 1999-2020	−3.13 (−4.35 to 10.61)	.40	−8.56 (−22.78 to 5.66)	.23	0.72 (−7.20 to 8.64)	.857
Difference with White, 1999-2004	11.48 (4.74 to 18.22)	.001	12.72 (7.10 to 18.33)	<.001	NA	NA
Difference with White, 2017-2020	13.89 (3.17 to 24.61)	.01	3.43 (−10.96 to 17.83)	.63	NA	NA
Absolute change in difference with White, 1999-2020	2.4 (−9.91 to 14.73)	.70	−9.28 (−24.26 to 5.69)	.22	NA	NA
Obesity						
Absolute change in prevalence, 1999-2020	3.64 (−9.28 to 16.56)	.57	9.26 (−9.83 to 28.36)	.33	12.08 (4.29 to 19.87)	.003
Difference with White, 1999-2004	15.07 (6.67 to 23.46)	<.001	1.19 (−10.69 to 13.07)	.84	NA	NA
Difference with White, 2017-2020	6.63 (−4.97 to 18.22)	.25	−1.63 (−14.17,10.91)	.79	NA	NA
Absolute change in difference with White, 1999-2020	−8.44 (−22.38 to 5.50)	.23	−2.82 (−19.70 to 14.06)	.74	NA	NA
Current smoking						
Absolute change in prevalence, 1999-2020	7.30 (4.06 to 18.66)	.20	−27.13 (−43.14 to −11.12)	.002	−2.28 (−8.75 to 4.19)	.484
Difference with White, 1999-2004	3.92 (−3.84 to 11.67)	.31	9.88 (−1.52 to 21.27)	.09	NA	NA
Difference with White, 2017-2020	13.50 (3.90 to 23.11)	.008	−14.97 (−22.35 to −7.59)	<.001	NA	NA
Absolute change in difference with White, 1999-2020	9.58 (−2.46 to 21.63.)	.12	−24.85 (−38.19 to −11.51)	<.001	NA	NA

Abbreviations: ACEI, angiotensin-converting enzyme inhibitor; ARB, angiotensin receptor blocker; NA, not applicable.

Data on aspirin use and optimal regimen were available from 2011 to 2020. In 2011-2012, the age-adjusted rates of aspirin use and optimal regimen were 74.5% (95% CI, 63.9%-85.2%) and 48.6% (95% CI, 36.4%-60.9%) for Black individuals, 61.4% (95% CI, 50.4%-72.5%) and 36.8% (95% CI, 24.3%-49.4%) for Hispanic and Latino individuals, and 77.8% (95% CI, 71.8%-83.7%) and 53.1% (95% CI, 46.8%-59.4%) for White individuals (Figure 2). Over the following 9 years, there was a 15.65 percentage points increase in aspirin use among Hispanic and Latino individuals (95% CI, 0.91-32.21 percentage points; *P* = .06) (Table 3). However, no significant improvement was observed in aspirin use and optimal regimen for any other racial or ethnic subgroup. In 2017-2020, optimal regimens were observed in 47.4% (95% CI, 39.3%-55.4%), 48.7% (95% CI, 36.7%-60.6%), and 53.0% (95% CI, 45.6%-60.4%) of Black, Hispanic and Latino, and White individuals, respectively. The difference in aspirin use and optimal regimen between White and Black individuals or between White individuals and Hispanic and Latino individuals did not significantly change during this period.

eFigure 2 in Supplement 1 shows trends in modifiable risk factors such as physical activity, obesity, and current smoking. Insufficient physical activity was prevalent across all racial and ethnic subgroups, with no significant change from 1999 to 2020 (Table 2). Obesity significantly increased for White individuals from 1999 to 2020 (37.5%; 95% CI, 33.2%-41.7% vs 49.9%; 95% CI, 44.1%-55.7%; *P* = .003), while it did not change significantly for Black individuals and Hispanic and Latino individuals. One encouraging trend is the improvement in smoking rates among Hispanic individuals, with a significant decrease in smoking rates, from 29.8% (95% CI, 14.1%-45.4%) to 3.8% (95% CI, 0%-8.7%) (*P* = .002). The rate also decreased for White individuals (25.0%; 95% CI, 21.4%-28.6% vs 22.9%; 95% CI, 18.4%-27.3%; *P* = .002), but not for Black individuals. The racial and ethnic differences between White individuals and other subgroups remained unchanged for insufficient physical activity, obesity, and smoking. In the 2017-2020 interval, compared with White individuals, Black individuals had higher rates of insufficient physical activity (13.9 percentage points; 95% CI, 3.2 to 24.6 percentage points; *P* = .01) and current smoking (13.5 percentage points; 95% CI, 3.9 to 23.1 percentage points; *P* = .008); rates for obesity were also higher, although these results were not significant (6.6 percentage points; 95% CI, −4.9 to 18.2 percentage points; *P* = .25). Hispanic and Latino individuals had similar rates of insufficient physical activity and obesity, but a lower rate of current smoking (−15.0 percentage points; 95% CI, −22.35 to −7.59 percentage points; *P* < .001) than White individuals.

### Trends in Social Determinants of Health by Race and Ethnicity

eFigure 3 in Supplement 1 displays trends in social determinants of health. Between 1999 and 2020, the percentage of individuals with less than a high school education decreased significantly among Black, Hispanic and Latino, and White individuals. Other social determinants, including family income, marital status, employment, and health insurance, did not exhibit significant changes across groups. Racial disparities in education levels remained consistent throughout the study period, with Hispanic and Latino individuals having the highest rates of education levels below high school. From 1999 to 2020, Black individuals consistently maintained lower levels of education, lower family income, and a higher likelihood of being single or living alone compared with their White counterparts. Additionally, Hispanic and Latino individuals consistently demonstrated the highest rate of education levels below high school throughout the study period.

## Discussion

In this 21-year assessment of ASCVD prevention care in the US, we found improvements in certain health care aspects. Notably, cholesterol control and use of statin and ACEI or ARB improved across racial and ethnic groups, and smoking cessation improved among Hispanic and Latino individuals. Despite progress, racial and ethnic disparities persisted. However, the most significant revelation is the substantial gap between current health care standards and optimal care, surpassing any differences observed among demographic groups. The finding that only 50% of patients, regardless of their racial and ethnic backgrounds, adhere to optimal prevention strategies underscores a significant opportunity for improvement. By ensuring that each individual receives the optimal care they require, we can collectively position ourselves for better outcomes, and this approach will also contribute to mitigating disparities. Overall, our findings emphasize the need for sustained efforts to bridge these gaps and optimize ASCVD prevention care for all patients.

Our study’s findings need to be contextualized within the broader landscape of health and health care disparities. First, this study reveals racial and ethnic disparities in the utilization of guideline-recommended therapies, which may be attributed to racial and ethnic differences in timely access to and affordability of health care. Black individuals and Hispanic and Latino individuals encounter higher rates of uninsurance, lack a regular source of care, and often delay seeking medical treatment compared with their White counterparts.^21,22,23,24,25,26,27^

These disparities are further compounded by social determinants of health, including factors such as education, employment, and social support, which disproportionately affect minoritized and low-income populations.^28,29,30^ For instance, individuals with limited educational or employment opportunities often contend with financial challenges, making it arduous to afford essential medications or attend medical appointments regularly. Moreover, the absence of robust social support networks can hinder individuals’ commitment to their prescribed treatment plans.^31^ The imperative lies in recognizing and actively addressing these disparities to propel the cause of health equity.

Furthermore, clinical inertia, where health care professionals hesitate to initiate or intensify therapy, may contribute to suboptimal ASCVD care.^32^ This inaction may derive from various causes, including concerns about adverse effects, patient adherence, or a perceived lack of urgency.^33^ The complex interplay of clinical guidelines, patient preferences, and health care system constraints can further exacerbate clinical inertia.

Lastly, the cardiovascular disease prevention guidelines over the past 2 decades have evolved.^2,16,17^ These guideline updates span various facets of cardiovascular care, with a notable shift toward personalized risk assessments and the introduction of new medications, such as *PCSK9* inhibitors and novel anticoagulants. These changes collectively aim to enhance the effectiveness of cardiovascular prevention efforts. Future research endeavors should evaluate whether these guideline revisions have been consistently implemented across racial and ethnic groups, thereby shedding light on the persistence of cardiovascular care disparities despite these advancements.

This study has important implications for both policy and clinical practice. First, our findings highlight the need to investigate the factors behind improvements in certain areas, such as smoking cessation in Hispanic and Latino individuals and cholesterol control in all subgroups. Addressing these factors can serve as models for enhancing health care outcomes more broadly. Moreover, despite these improvements, the revelation of a substantial gap between current care and optimal care affecting patients across demographic groups underscores a critical area for improvement. Sustained efforts are needed to address these disparities and elevate the overall quality of ASCVD prevention care. There needs to be a concerted effort to ensure that health care services and interventions for ASCVD prevention are accessible and affordable to all, regardless of their socioeconomic status, race, or ethnicity. Furthermore, health care delivery models should prioritize personalized and optimal care for each patient. This means tailoring treatment plans to individual patient needs, considering their unique risk factors and preferences. Implementing patient-centered care models that emphasize shared decision-making and culturally sensitive approaches can lead to better engagement and adherence to preventive measures.^34^ Additionally, investments in health information technology can facilitate the delivery of personalized care. Electronic health records can help clinicians track and manage patients’ cardiovascular health more effectively, ensuring that interventions are timely and appropriate.^35^ Telemedicine and remote monitoring can also extend care to underserved or remote communities, reducing geographical disparities in access to health care.^36^ In the end, the collaborative efforts of policymakers, health care professionals, and community organizations are crucial in addressing cardiovascular health disparities comprehensively.

This study extends existing research in several critical ways. First, it uses the most recent nationally representative data to investigate the use of guideline-recommended therapies for secondary prevention. This approach extends the findings of earlier studies, such as Massing et al^11^ and Shah et al,^12^ by providing a more up-to-date and comprehensive picture of disparities in medication use among patients with ASCVD. Moreover, the study goes beyond previous work by analyzing a wide range of medications used for secondary prevention, including individual medication groups and optimal regimens. By doing so, it offers a more detailed and nuanced understanding of medication utilization patterns among ASCVD patients. Finally, the study provides quantitative data on lifestyle modifications (eg, weight loss, physical activity, and smoking cessation) recommended for secondary prevention, which are crucial for reducing the risk of ASCVD.^2^

### Limitations

Our study has several limitations. First, we relied on self-reported history of ASCVD to define the secondary prevention population, which may introduce measurement error. Nonetheless, previous studies have shown that self-reported myocardial infarction has high sensitivity and specificity (over 97%),^37^ suggesting that self-report could be a valid instrument to define the study population in this analysis. Second, while this study focuses on individuals with established ASCVD, the distinction is often based on whether diagnostic testing is performed, or events are recognized. Therefore, a secondary prevention cohort is likely specific for people with established coronary disease but may not be sensitive. Third, the data on medication use was self-reported, which may be subject to recall bias. To minimize this bias, we combined self-report medication use with information gathered during the actual examination of the patient’s prescription medication. Fourth, the sample size of participants with ASCVD by race and ethnicity in each NHANES cycle is relatively small, which may limit the power to detect changes for certain therapies. Finally, because evidence and clinical guidelines were evolving during the study period, some of the change in utilization of guideline-recommended therapies over time may reflect the evolution of the knowledge base rather than actual changes in clinical practice.

## Conclusions

In this cross-sectional study of US adults with ASCVD, significant disparities have persisted between current care and optimal care, surpassing any differences observed among demographic groups. Our findings highlight the critical need for sustained efforts to bridge these gaps and achieve better outcomes for all patients, regardless of their racial and ethnic backgrounds.

## Notes

Supplement 1.
eMethods.
**eTable 1.** Definition of Sociodemographic, Behavioral, and Clinical Variables in NHANES **eTable 2. **Secondary Prevention and Risk Reduction Therapy for Patients with Atherosclerotic Cardiovascular Disease **eTable 3. **Lifestyle and Comorbidity Characteristics of Adults Reporting Prior Atherosclerotic Cardiovascular Disease in 1999-2020 **eTable 4. **Cholesterol Trends in NHANES Participants Reporting Prior Atherosclerotic Cardiovascular Disease, by Race and Ethnicity **eTable 5. **Blood Pressure Trends in NHANES Participants Reporting Prior Atherosclerotic Cardiovascular Disease, by Race and Ethnicity **eTable 6. **Blood Glucose Trends in NHANES Participants Reporting Prior Atherosclerotic Cardiovascular Disease, by Race and Ethnicity **eFigure 1. **Study Population Flowchart **eFigure 2. **Trends in Modifiable Lifestyle Risk Factors Among Adults with Atherosclerotic Cardiovascular Disease by Race and Ethnicity, 1999-2020 **eFigure 3. **Trends in Social Determinants of Health Among Adults with Atherosclerotic Cardiovascular Disease by Race and Ethnicity, 1999-2020

Supplement 2.
Data Sharing Statement
