# Supplementary material for: National Trends in Racial and Ethnic Disparities in Use of Recommended Therapies in Adults with Atherosclerotic Cardiovascular Disease, 1999-2020
Source: JAMA Netw Open. 2023 Dec 1;6(12):e2345964. doi: 10.1001/jamanetworkopen.2023.45964 (PMC10692850; doi:10.1001/jamanetworkopen.2023.45964)
Supplement: Supplement 1. — eMethods. eTable 1. Definition of Sociodemographic, Behavioral, and Clinical Variables in NHANES eTable 2. Secondary Prevention and Risk Reduction Therapy for Patients with Atherosclerotic Cardiovascular Disease eTable 3. Lifestyle and Comorbidity Characteristics of Adults Reporting Prior Atherosclerotic Cardiovascular Disease in 1999-2020 eTable 4. Cholesterol Trends in NHANES Participants Reporting Prior Atherosclerotic Cardiovascular Disease, by Race and Ethnicity eTable 5. Blood Pressure Trends in NHANES Participants Reporting Prior Atherosclerotic Cardiovascular Disease, by Race and Ethnicity eTable 6. Blood Glucose Trends in NHANES Participants Reporting Prior Atherosclerotic Cardiovascular Disease, by Race and Ethnicity eFigure 1. Study Population Flowchart eFigure 2. Trends in Modifiable Lifestyle Risk Factors Among Adults with Atherosclerotic Cardiovascular Disease by Race and Ethnicity, 1999-2020 eFigure 3. Trends in Social Determinants of Health Among Adults with Atherosclerotic Cardiovascular Disease by Race and Ethnicity, 1999-2020 [file jamanetwopen-e2345964-s001.pdf]

Supplemental Online Content

Lu Y, Liu Y, Dhingra LS, et al. National Trends in Racial and Ethnic Disparities in Use of Recommended Therapies in Adults with Atherosclerotic Cardiovascular Disease, 1999-2020. *JAMA Netw Open.* 2023;6(12):e2345964.
doi:10.1001/jamanetworkopen.2023.45964

eMethods.

eTable 1. Definition of Sociodemographic, Behavioral, and Clinical Variables in NHANES

eTable 2. Secondary Prevention and Risk Reduction Therapy for Patients with Atherosclerotic Cardiovascular Disease

eTable 3. Lifestyle and Comorbidity Characteristics of Adults Reporting Prior Atherosclerotic Cardiovascular Disease in 1999-2020

eTable 4. Cholesterol Trends in NHANES Participants Reporting Prior Atherosclerotic Cardiovascular Disease, by Race and Ethnicity

eTable 5. Blood Pressure Trends in NHANES Participants Reporting Prior Atherosclerotic Cardiovascular Disease, by Race and Ethnicity

eTable 6. Blood Glucose Trends in NHANES Participants Reporting Prior Atherosclerotic Cardiovascular Disease, by Race and Ethnicity

eFigure 1. Study Population Flowchart

eFigure 2. Trends in Modifiable Lifestyle Risk Factors Among Adults with Atherosclerotic Cardiovascular Disease by Race and Ethnicity, 1999-2020

eFigure 3. Trends in Social Determinants of Health Among Adults with Atherosclerotic Cardiovascular Disease by Race and Ethnicity, 1999-2020

This supplemental material has been provided by the authors to give readers additional information about their work.
.

© 2023 Lu Y et al. *JAMA Network Open.*

eMethods.

Data Collection in NHANES

NHANES is a series of cross-sectional, multistage, weighted surveys that provide nationally representative estimates for the non-institutionalized US population.[1] In-home interviews are conducted with all participants, and a random subsample of participants also undergo standardized physical examinations and provide blood and urine specimens for laboratory testing.[2]

During the in-home interview, detailed information on participants' demographics, socioeconomic status, and medical history was collected. Demographic and socioeconomic variables included age, sex, education level, family income (based on the percent of family income relative to the federal poverty limit from the Census Bureau), insurance status, marital status, employment status, smoking status, and alcohol intake. Physical activity was categorized into three groups - inactive (no participation or fewer than 10 minutes of moderate or vigorous physical activity per week), insufficient (between 10-149 minutes per week of moderate physical activity or between 10-74 minutes per week of vigorous physical activity), and recommended (150 minutes or more of moderate physical activity or 75 minutes or more of vigorous physical activity per week) - based on established physical activity guidelines.[3, 4] The definitions of these covariates are provided in Supplementary Table S1. We also obtained information on the use of medication, including aspirin, statin, angiotensin-converting enzyme inhibitor (ACEI), and angiotensin II receptor blocker (ARB), by self-report and review of participant prescription medication bottles.

Physical examinations were conducted by trained staff who measured participants' weight and height to calculate body mass index (BMI), with obesity defined as a BMI ≥30 kg/m^2. Blood pressure was measured using a mercury sphygmomanometer after the participant rested quietly in a seated position for at least 5 minutes. Three blood pressure measurements were obtained, and the mean of all measurements was used in analyses. Blood samples were collected at the mobile examination center and then sent to central laboratories for analysis. Standard methods were used to determine total cholesterol, high-density lipoprotein (HDL) cholesterol, low-density lipoprotein (LDL) cholesterol, triglycerides, plasma glucose, and hemoglobin A1c. Fasting blood samples collected over an eight-hour period were available in a subsample of survey participants for measuring LDL cholesterol, triglycerides, and glucose.

Reference:

1. Centers for Disease Control and Prevenetion. US National Health and Nutrition Examination Survey.http://www.cdc.gov/nchs/nhanes.htm. Accessed on April 17, 2017.
2. Centers for Disease Control and Prevenetion. National Health and Nutrition Examination Survey: Plan and Operations, 1999–2010.https://www.cdc.gov/nchs/data/series/sr_01/sr01_056.pdf. Accessed on August 17, 2021.
3. Haskell WL, Lee I-M, Pate RR, Powell KE, Blair SN, Franklin BA, Macera CA, Heath GW, Thompson PD and Bauman A. Physical activity and public health: updated recommendation for adults from the American College of Sports Medicine and the American Heart Association. *Circulation*. 2007;116:1081.
4. Physical Activity Guidelines Advisory Committee. Physical activity guidelines advisory committee report, 2008. *Washington, DC: US Department of Health and Human Services*. 2008;2008:A1-H14.

© 2023 Lu Y et al. *JAMA Network Open.*

eTable 1. Definition of Sociodemographic, Behavioral, and Clinical Variables in NHANES

**Risk factor**	**Ascertainment in NHANES**	**Definition of variables used in analysis**
Marital status	In-person interview: Please describe your current marital status.	Marital status is classified as married/ living with partners vs. others (widowed, divorced, or separated; and never married).
Highest education level	In-person interview: What is the highest grade or level of school you have completed or the highest degree you have received?	Highest education level is classified as less than high school, high school, greater than high school.
Family income	In-person interview: Please describe your family income (reported as a range value in dollars)	Based on percent of family income relative to the federal poverty limit from the Census Bureau, family income is categorized as high/middle income (≥200%] and low-income (<200%]
Health insurance	In-person interview: Are you covered by health insurance or some other kind of health care plan? [Include health insurance obtained through employment or purchased directly as well as government programs like Medicare and Medicaid that provide medical care or help pay medical bills.]	Individuals are classified as insured if they had any private health insurance, Medicare, Medicaid, military plan, government or state-sponsored health plan.
Smoker status	In-person interview:<br>• Ever smoke cigarettes in entire life<br>• Do you now smoke cigarettes?	Never smokers are defined as individuals who stated never smokes.<br>Former smokers are defined as individuals who stated that they had smoked but now did not smoke.<br>Current smokers are defined as individuals who stated that they smoked cigarettes currently.
Alcohol intake	In-person interview:<br>• Ever had a drink of any kind of alcohol<br>• For the Past 12 months, how often do you have alcohol drink<br>• For the past 12 months the average number of alcohol drinks per day	Never drinkers are defined as individuals who stated never had any kind of alcohol drink. Former drinkers are defined as individuals who stated they had had e drinks but did not drink for the past 12 months. Light drinkers are defined as individuals who self-reported 0-0.5 average daily drinks. Moderate drinkers are defined as individuals who self-reported (0.5-1.5 for women; 0.5-2.5 for men) average daily drinks. Heavy drinkers are defined as individuals who self-reported (1.5+ for women; 2.5+ for men) average daily drinks.

© 2023 Lu Y et al. *JAMA Network Open.*

Physical activity	In-person interview:<br>• Behavioral Risk Factor Surveillance Survey (BRFSS) physical activity instrument.<br>• Patients were asked if they participated in moderate or vigorous physical activity during the past 30 days. If they answered yes to either question, they were then asked the duration and frequency of their participation in physical activity for an average week.	Physical activity is classified into three levels: recommended (150 min or more of moderate physical activity or 75 min or more of vigorous physical activity per week), insufficient (between 10-149 min per week of moderate physical activity or between 10-74 min per week of vigorous physical activity), or inactive (no participation or fewer than 10 min of moderate or vigorous physical activity per week).
Obesity	All participants ages 2 and older had their standing height and weight measured during their physical examination.	Obesity is defined as body mass index (BMI)≥ 30kg/m^2, where BMI is calculated as weight divided by the square of height.
Diabetes	In-person interview:<br>• Are you now taking diabetic pills to lower your blood sugar? These are sometimes called oral agents or oral hypoglycemic agents.<br>• Are you now taking insulin?<br>• Self-reported use of prescription medications during a one-month period prior to the survey date.<br><br>Lab measurement:<br>All participants ages 12 and older are given the option of a HbA1C% test during their physical examination. Besides, Participants aged 12 years and older who were examined in the morning session were tested for fasting glucose.	Diabetes is defined based on fasting glucose >= 126 mg/dL, or HbA1c ≥ 6.5%, or currently on antidiabetic medication.
Hypertension	In-person interview:<br>Are you now taking prescribed medicine for high blood pressure (BP)<br><br>Examination measurement:<br>Blood pressure (BP) is measured on all examinees 8 years and older.	Hypertension is defined as average systolic blood pressure >=140, or averaged diastolic blood pressure>=90, or currently on antihypertensive medication.
Chronic kidney disease	Lab measurement:<br>• All Participants aged 12 years and older are tested creatinine, which can be applied to estimate	Chronic kidney disease is defined as estimated glomerular filtration rate (eGFR) < 60 ml/min/1.73m^2, or urinary albumin-to-creatinine ratio >= 30mg/g.

© 2023 Lu Y et al. *JAMA Network Open.*

	glomerular filtration rate (GFR) using CKD-EPI creatinine equation.<br>• Urinary albumin and urinary creatinine are measured in a random urine collected in the MEC. Participants aged 6 years and older were eligible to be tested.	
History of hyperlipidemia	In-person interview:<br>• Has a doctor or other health professional ever told you that you had high cholesterol?	History of hyperlipidemia is defined based on self-reported previous physician diagnosis of high cholesterol.
History of MI/stroke	In-person interview:<br>• Has a doctor or other health professional ever told you that you had heart attack?<br>• Has a doctor or other health professional ever told you that you stroke?	History of MI/stroke is defined based on self-reported previous physician diagnosis of heart attack or stroke.
History of cancer	In-person interview:<br>• Has a doctor or other health professional ever told you that you had cancer?	History of cancer is defined based on self-reported previous physician diagnosis of cancer.

© 2023 Lu Y et al. *JAMA Network Open.*

eTable 2. Secondary Prevention and Risk Reduction Therapy for Patients with Atherosclerotic Cardiovascular Disease

Area for intervention	Guideline recommendation or treatment goal	Strength of recommendation
**Pharmacological medications**		
Aspirin	Aspirin 75–162 mg daily is recommended in all patients with CAD unless otherwise contraindicated	I(A)
Statin	High-intensity Statin therapy should be started in the absence of contraindications or documented adverse effects	I(A)
Beta blocker	Beta-Blocker therapy should be used in all patients with left ventricular systolic dysfunction (ejection fraction <40%) with heart failure or prior myocardial infarction, unless contraindicated.	I(A)
ACEI	ACE inhibitors should be started and continued indefinitely in all patients with left ventricular ejection fraction <=40% and in those with hypertension, diabetes, or chronic kidney disease, unless contraindicated.	I(A)
**Cardiovascular risk factors**		
Lipid management	Goal: Treatment with statin therapy; use statin therapy to achieve an LDL-C of <100 mg/dL; for very high-risk patients an LDL-C <70 mg/dL is reasonable; if triglycerides are >= 200 mg/dL, non–HDL-C should be <130 mg/dL, whereas non–HDL-C <100 mg/dL for very high-risk patients is reasonable.	
Blood pressure control	Goal: <140/90 mm Hg	
Diabetes management	Goal: Target HbA1c <=7%	
**Lifestyle modification**		
Weight management	Goals: Body mass index: 18.5 to 24.9 kg/m^2. Waist circumference: women <35 inches (<89 cm), men <40 inches (<102 cm)	
Physical activity	Goal: At least 30 minutes, 7 days per week (minimum 5 days per week)	
Smoking cessation	Goal: Complete cessation. No exposure to environmental tobacco smoke.	

© 2023 Lu Y et al. *JAMA Network Open.*

eTable 3. Lifestyle and Comorbidity Characteristics of Adults Reporting Prior Atherosclerotic Cardiovascular Disease in 1999-2020

Characteristics, % (95% CI)	Non-Hispanic White individuals	Non-Hispanic Black individuals	Hispanic/Latino individuals
**Smoking Status**			
Current smoker	625 (22.5%, [20.5-24.6])	361 (33.6%, [30.3-36.8])	155 (19.9%, [14.8-24.9])
Former smoker	1349 (40.4%, [38.3-42.5])	364 (27.3%, [24.3-30.4])	347 (32.3%, [27.9-36.7])
Never smoker	1142 (37.1%, [34.6-39.5])	443 (39.1%, [36.1-42.2])	427 (47.8%, [42.7-53])
**Physical activity**			
Recommended	720 (27.1%, [24.9-29.3])	188 (16%, [13.4-18.6])	177 (20.8%, [16.7-24.8])
Inactive	1991 (57.9%, [55.4-60.4])	830 (70.9%, [67.5-74.2])	659 (69%, [64.4-73.5])
Insufficient	402 (15%, [13.3-16.7])	152 (13.2%, [10.8-15.5])	92 (10.3%, [7.6-13])
**Alcohol intake**			
Never	303 (20.5%, [17.6-23.4])	114 (23.1%, [18.7-27.6])	132 (28.1%, [21.9-34.4])
Former	258 (16.6%, [14.2-19])	120 (23.3%, [19.4-27.2])	71 (13.5%, [8.6-18.5])
Light drinker	397 (25.3%, [22.3-28.3])	94 (19%, [14.4-23.6])	56 (11.5%, [7.4-15.6])
Moderate drinker	376 (28.4%, [25.3-31.5])	104 (18.1%, [13.5-22.8])	92 (19.6%, [14-25.2])
Heavy drinker	113 (9.2%, [7.1-11.2])	75 (16.4%, [13-19.8])	94 (27.2%, [19.9-34.6])
**BMI, kg/m2**			
<25	654 (21.5%, [19.9-23.1])	231 (21.6%, [18.8-24.5])	136 (16.2%, [12.6-19.8])
25-<30	986 (35.3%, [33.2-37.3])	287 (26%, [22.6-29.4])	308 (36.9%, [32.2-41.7])
>=30	1070 (43.2%, [40.9-45.6])	528 (52.3%, [48.3-56.4])	370 (46.9%, [41.7-52.1])
**Medical history**			
Diabetes	725 (27.1%, [25.1-29])	374 (36%, [32.5-39.6])	552 (36.8%, [31.8-41.9])
Hyperlipidemia	415 (66.2%, [64-68.5])	520 (60.8%, [57.3-64.2])	318 (57.6%, [52.3-63])
Hypertension	200 (67.5%, [65.4-69.5])	657 (82.1%, [79.6-84.7])	272 (69.1%, [63.9-74.3])
Cancer	1008 (25.5%, [23.4-27.5])	91 (12%, [9.6-14.3])	838 (9.9%, [6.7-13.2])

Abbreviations: BMI: body mass index; CI: confidence interval; SD: standard deviation.

© 2023 Lu Y et al. *JAMA Network Open.*

eTable 4. Cholesterol Trends in NHANES Participants Reporting Prior Atherosclerotic Cardiovascular Disease, by Race and Ethnicity

	**1999-2004**	**2005-2008**	**2009-2012**	**2013-2016**	**2017-2020**	**P trend**
**Overall, N (total)**	1473	1016	947	924	858	
Treated (% of total)	50.3	62.5	67.5	68.3	72.7	0.017
Controlled (% of treated)	61.9	82.2	78.3	84.5	83.2	0.125
Total cholesterol, M (SD)	189.8 (39.4)	169.3 (37.3)	170.8 (38.2)	164.1 (37.1)	162.7 (37.6)	0.050
HDL, M (SD)	46.4 (12.2)	48.7 (12.8)	47.1 (12.9)	47.8 (14.1)	49.5 (13.2)	0.249
LDL, M (SD)	106.0 (29.6)	90.8 (34.7)	95.1 (32.3)	84.3 (31.7)	86.5 (31.7)	0.054
Untreated (% of total)	49.7	37.5	32.4	31.7	27.3	0.017
At goal (% of untreated)	46.0	51.2	48.6	47.7	48.9	0.630
Total cholesterol, M (SD)	207.6 (39.6)	201.1 (40.1)	197.8 (39.2)	197.7 (36.5)	198.7 (38.0)	0.056
HDL, M (SD)	47.9 (13.0)	50.2 (13.4)	49.3 (13.9)	51.2 (13.5)	49.1 (13.2)	0.368
LDL, M (SD)	121.8 (32.1)	117.4 (33.7)	117.2 (32.7)	121.0 (31.8)	122.4 (35.6)	0.826
**Black, N (total)**	261	217	227	216	249	
Treated (% of total)	39.3	54.7	60.8	67.8	57.4	0.142
Controlled (% of treated)	61.0	71.8	76.5	85.9	86.0	0.006
Total cholesterol, M (SD)	193.4 (38.4)	179.8 (42.6)	176.0 (36.7)	167.4 (39.5)	163.9 (36.9)	0.003
HDL, M (SD)	47.4 (13.7)	51.6 (12.9)	49.8 (13.2)	51.2 (13.6)	51.8 (12.1)	0.203
LDL, M (SD)	115.7 (36.2)	95.3 (32.2)	102.6 (31.2)	95.2 (34.4)	80.5 (26.5)	0.067
Untreated (% of total)	60.7	45.3	39.2	32.2	42.6	0.142
At goal (% of untreated)	49.2	44.7	62.7	56.4	60.1	0.188

© 2023 Lu Y et al. *JAMA Network Open.*

Total cholesterol, M (SD)	204.3 (41.6)	196.8 (39.9)	184.5 (39.0)	189.7 (39.9)	188.0 (38.8)	0.089
HDL, M (SD)	52.2 (13.9)	53.3 (13.7)	50.5 (14.7)	53.2 (14.1)	51.9 (13.7)	0.914
LDL, M (SD)	112.9 (35.4)	106.7 (41.1)	116.0 (32.9)	114.7 (37.7)	119.5 (39.4)	0.190
**Hispanic, N (total)**	258	153	177	205	137	
Treated (% of total)	40.0	59.9	60.5	65.3	64.2	0.105
Controlled (% of treated)	68.6	73.8	66.3	80.5	88.7	0.082
Total cholesterol, M (SD)	189.8 (46.9)	176.2 (44.2)	181.4 (47.8)	168.4 (40.1)	157.5 (28.6)	0.024
HDL, M (SD)	43.7 (9.9)	44.5 (11.1)	46.5 (12.2)	45.5 (11.2)	50.2 (12.1)	0.111
LDL, M (SD)	110.9 (44.3)	86.1 (37.7)	94.7 (32.5)	98.4 (39.6)	85.5 (25.1)	0.318
Untreated (% of total)	60.0	40.1	39.5	34.7	35.8	0.105
At goal (% of untreated)	50.2	44.3	40.3	48.6	62.5	0.400
Total cholesterol, M (SD)	200.1 (36.7)	193.6 (34.7)	202.0 (35.0)	190.3 (34.9)	183.5 (33.5)	0.122
HDL, M (SD)	45.6 (10.4)	47.8 (13.6)	50.1 (12.9)	46.5 (12.1)	48.6 (13.3)	0.375
LDL, M (SD)	116.7 (32.6)	114.4 (27.7)	107.8 (36.4)	113.0 (26.5)	110.4 (29.6)	0.248
**White, N (total)**	954	646	543	503	472	
Treated (% of total)	52.9	64.4	70.2	69.5	76.4	0.016
Controlled (% of treated)	61.8	83.8	79.8	84.9	82.7	0.180
Total cholesterol, M (SD)	189.6 (39.2)	167.9 (36.3)	169.4 (37.3)	163.3 (36.5)	163.0 (38.3)	0.066
HDL, M (SD)	46.5 (12.1)	48.7 (12.9)	46.8 (12.9)	47.5 (14.4)	49.2 (13.4)	0.356
LDL, M (SD)	105.1 (28.4)	90.7 (34.8)	94.4 (32.4)	81.8 (24.6)	87.2 (32.6)	0.061
Untreated (% of total)	47.1	35.6	29.8	30.5	23.6	0.016
At goal (% of untreated)	44.6	53.1	46.4	45.0	43.2	0.668

© 2023 Lu Y et al. *JAMA Network Open.*

Total cholesterol, M (SD)	208.9 (39.4)	202.8 (40.6)	199.9 (39.5)	200.7 (35.6)	204.5 (37.3)	0.228
HDL, M (SD)	47.5 (12.9)	49.9 (13.3)	48.8 (13.9)	51.7 (13.5)	48.4 (13.0)	0.345
LDL, M (SD)	123.8 (31.3)	119.8 (32.8)	118.8 (32.2)	125.1 (31.3)	126.8 (34.9)	0.594

© 2023 Lu Y et al. *JAMA Network Open.*

eTable 5. Blood Pressure Trends in NHANES Participants Reporting Prior Atherosclerotic Cardiovascular Disease, by Race and Ethnicity

	**1999-2004**	**2005-2008**	**2009-2012**	**2013-2016**	**2017-2020**	**P trend**
**Overall, N (total)**	1473	1016	947	924	858	
Treated (% of total)	76.1	79.5	79.8	81.4	78.3	0.313
Controlled (% of treated)	61.6	64.7	70.4	75.6	68.4	0.121
Systolic BP, M (SD)	134.1 (22.4)	131.6 (21.1)	130.3 (19.9)	128.8 (19.0)	130.9 (20.8)	0.093
Diastolic BP, M (SD)	68.8 (12.7)	67.5 (12.5)	66.5 (12.1)	66.5 (12.0)	72.2 (12.0)	0.698
Untreated (% of total)	23.9	20.5	20.2	18.6	21.7	0.313
At goal (% of untreated)	67.9	64.1	79.2	70.5	69.1	0.638
Systolic BP, M (SD)	129.2 (20.3)	126.3 (18.7)	123.0 (17.2)	124.6 (18.8)	129.4 (15.6)	0.863
Diastolic BP, M (SD)	73.6 (10.9)	72.1 (11.5)	70.1 (11.5)	70.4 (10.2)	76.1 (12.1)	0.763
**Black, N (total)**	261	217	227	216	249	
Treated (% of total)	82.5	84.5	87.0	84.3	82.1	0.935
Controlled (% of treated)	45.0	52.7	62.3	62.5	57.0	0.139
Systolic BP, M (SD)	139.4 (21.5)	136.8 (23.4)	131.8 (19.5)	132.9 (21.1)	133.6 (22.6)	0.092
Diastolic BP, M (SD)	76.1 (14.2)	72.0 (13.0)	69.6 (13.8)	70.5 (13.7)	75.8 (13.2)	0.876
Untreated (% of total)	17.5	15.5	13.0	15.7	17.9	0.935
At goal (% of untreated)	52.0	35.0	65.6	49.8	46.3	0.989
Systolic BP, M (SD)	128.7 (25.1)	132.2 (18.4)	124.3 (20.3)	130.0 (21.5)	131.5 (17.8)	0.853

© 2023 Lu Y et al. *JAMA Network Open.*

Diastolic BP, M (SD)	74.7 (12.4)	78.6 (13.4)	72.5 (11.6)	71.4 (11.9)	78.6 (11.7)	0.967
**Hispanic, N (total)**	258	153	177	205	137	
Treated (% of total)	72.6	75.2	79.2	79.9	82.6	0.005
Controlled (% of treated)	56.1	48.6	62.5	60.4	58.6	0.392
Systolic BP, M (SD)	133.3 (24.0)	134.7 (19.2)	132.8 (23.8)	134.6 (19.5)	132.1 (22.7)	0.726
Diastolic BP, M (SD)	70.9 (11.7)	72.4 (11.3)	67.6 (13.3)	68.2 (13.2)	73.3 (11.7)	0.939
Untreated (% of total)	27.4	24.8	20.8	20.1	17.4	0.005
At goal (% of untreated)	65.5	62.5	73.5	67.5	56.9	0.804
Systolic BP, M (SD)	129.7 (20.2)	123.4 (15.1)	119.4 (16.9)	125.2 (17.9)	128.8 (19.6)	0.955
Diastolic BP, M (SD)	72.3 (10.8)	72.8 (12.5)	69.2 (9.0)	70.5 (11.7)	76.0 (11.1)	0.687
**White, N (total)**	954	646	543	503	472	
Treated (% of total)	75.4	79.1	78.7	81.2	77.2	0.363
Controlled (% of treated)	64.8	68.1	73.3	79.7	71.4	0.124
Systolic BP, M (SD)	133.4 (22.3)	130.7 (20.7)	129.8 (19.5)	127.6 (18.4)	130.5 (20.3)	0.123
Diastolic BP, M (SD)	67.8 (12.2)	66.6 (12.3)	66.0 (11.7)	65.6 (11.4)	71.6 (11.8)	0.623
Untreated (% of total)	24.6	20.9	21.3	18.8	22.8	0.363
At goal (% of untreated)	69.4	67.2	80.9	73.6	72.8	0.440
Systolic BP, M (SD)	129.2 (19.7)	125.8 (19.1)	123.5 (16.8)	123.5 (18.3)	129.1 (14.8)	0.781
Diastolic BP, M (SD)	73.6 (10.7)	71.1 (10.8)	69.9 (11.9)	70.2 (9.6)	75.7 (12.3)	0.781

© 2023 Lu Y et al. *JAMA Network Open.*

eTable 6. Blood Glucose Trends in NHANES Participants Reporting Prior Atherosclerotic Cardiovascular Disease, by Race and Ethnicity

	**1999-2004**	**2005-2008**	**2009-2012**	**2013-2016**	**2017-2020**	**P trend**
**Overall, N (total)**	1473	1016	947	924	858	
Treated (% of total)	19.9	25.5	26.6	28.6	32.6	0.006
Controlled (% of treated)	65.3	70.8	68.8	66.0	58.3	0.383
Fasting glucose, M (SD)	118.1 (31.6)	126.7 (24.1)	130.2 (25.5)	126.3 (23.1)	126.4 (21.0)	0.543
HbA1c, M (SD)	6.63 (0.70)	6.49 (0.73)	6.58 (0.69)	6.54 (0.70)	6.71 (0.63)	0.581
Untreated (% of total)	80.1	74.5	73.4	71.4	67.4	0.006
At goal (% of untreated)	99.4	98.7	98.7	97.8	99.5	0.977
Fasting glucose, M (SE)	99.7 (14.1)	100.6 (13.5)	99.9 (11.6)	104.9 (20.0)	102.4 (13.5)	0.190
HbA1c, M (SE)	5.44 (0.46)	5.50 (0.50)	5.59 (0.42)	5.63 (0.57)	5.63 (0.50)	0.006
**Black, N (total)**	261	217	227	216	249	
Treated (% of total)	36.3	41.5	35.6	36.3	38.2	0.858
Controlled (% of treated)	63.8	73.0	66.3	68.0	68.0	0.916
Fasting glucose, M (SD)	118.1 (25.6)	122.6 (24.9)	118.8 (28.6)	109.9 (25.4)	123.0 (27.5)	0.796
HbA1c, M (SD)	6.63 (0.80)	6.50 (0.74)	6.62 (0.80)	6.55 (0.70)	6.64 (0.64)	0.631
Untreated (% of total)	63.7	58.5	64.4	63.7	61.8	0.858
At goal (% of untreated)	99.1	92.2	98.2	96.2	98.5	0.757
Fasting glucose, M (SE)	93.4 (16.3)	109.6 (23.3)	90.1 (8.2)	104.2 (21.6)	103.1 (14.6)	0.449
HbA1c, M (SE)	5.35 (0.55)	5.76 (0.46)	5.66 (0.54)	5.64 (0.52)	5.70 (0.46)	0.256
**Hispanic, N (total)**	258	153	177	205	137	

© 2023 Lu Y et al. *JAMA Network Open.*

Treated (% of total)	31.3	44.3	34.9	42.5	39.9	0.489
Controlled (% of treated)	55.8	70.0	64.2	58.3	71.6	0.629
Fasting glucose, M (SE)	118.0 (23.6)	121.0 (21.2)	122.7 (26.8)	127.2 (29.3)	119.1 (18.5)	0.656
HbA1c, M (SE)	6.74 (0.59)	6.41 (0.78)	6.77 (0.66)	6.67 (0.74)	6.69 (0.72)	0.934
Untreated (% of total)	68.7	55.7	65.1	57.5	60.1	0.489
At goal (% of untreated)	97.7	99.6	97.0	97.4	98.9	0.735
Fasting glucose, M (SE)	98.2 (12.7)	107.0 (15.1)	100.7 (13.5)	99.4 (14.4)	103.1 (17.3)	0.870
HbA1c, M (SE)	5.42 (0.53)	5.23 (0.50)	5.77 (0.66)	5.53 (0.60)	5.59 (0.47)	0.364
**White, N (total)**	954	646	543	503	472	
Treated (% of total)	17.5	22.5	24.8	26.6	31.6	0.003
Controlled (% of treated)	66.1	70.2	69.7	66.4	55.3	0.324
Fasting glucose, M (SE)	118.2 (33.7)	128.3 (24.1)	132.6 (24.3)	129.4 (20.2)	128.4 (19.2)	0.504
HbA1c, M (SE)	6.62 (0.69)	6.50 (0.73)	6.55 (0.67)	6.52 (0.69)	6.73 (0.62)	0.535
Untreated (% of total)	82.5	77.5	75.2	73.4	68.4	0.003
At goal (% of untreated)	99.5	99.2	98.8	97.9	99.7	0.955
Fasting glucose, M (SE)	100.6 (13.8)	98.9 (11.6)	101.1 (11.3)	106.8 (21.0)	102.3 (12.9)	0.276
HbA1c, M (SE)	5.45 (0.44)	5.49 (0.50)	5.55 (0.34)	5.65 (0.58)	5.62 (0.51)	0.007

© 2023 Lu Y et al. *JAMA Network Open.*

eFigure 1. Study Population Flowchart

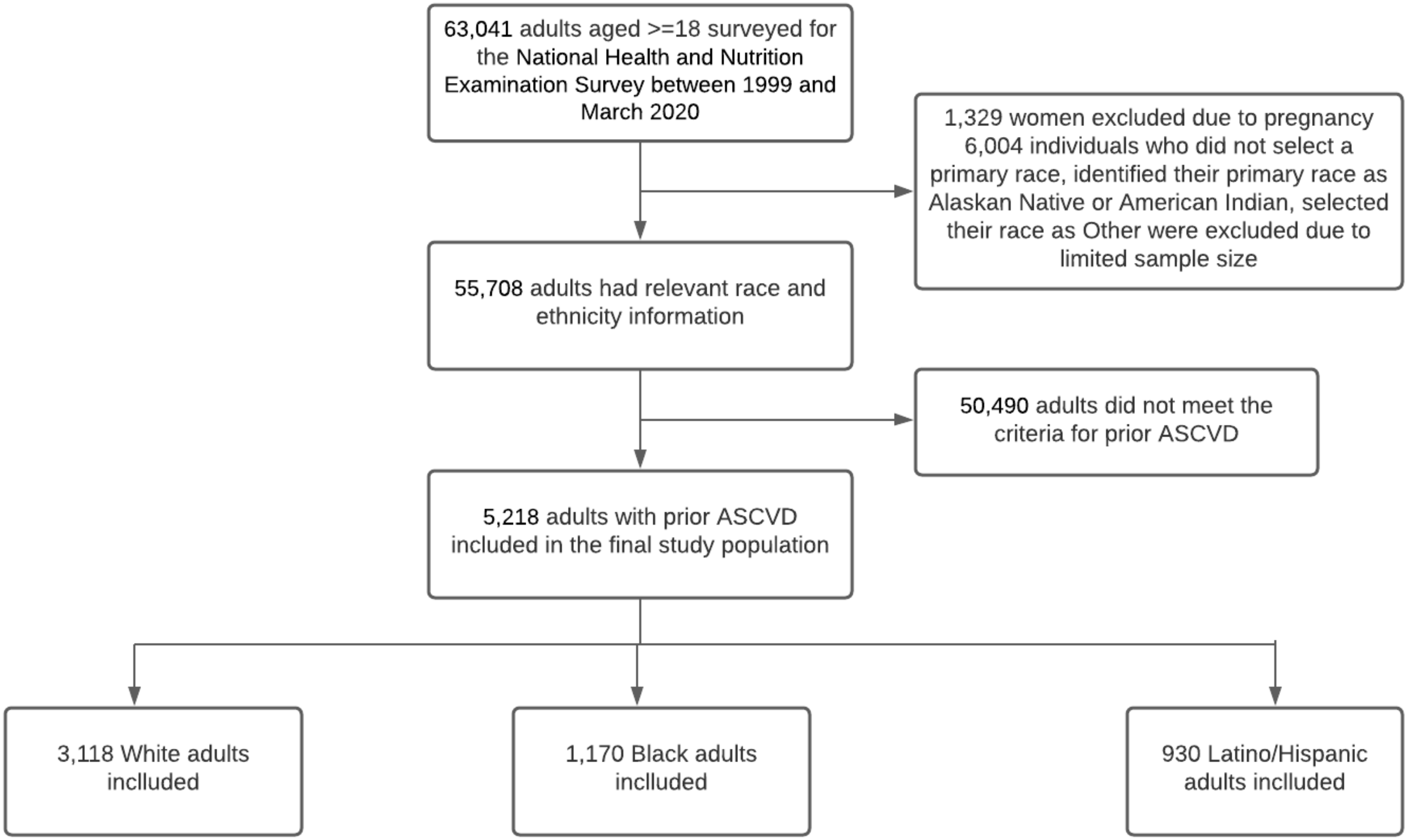

© 2023 Lu Y et al. *JAMA Network Open.*

eFigure 2. Trends in Modifiable Lifestyle Risk Factors Among Adults with Atherosclerotic Cardiovascular Disease by Race and Ethnicity, 1999-2020

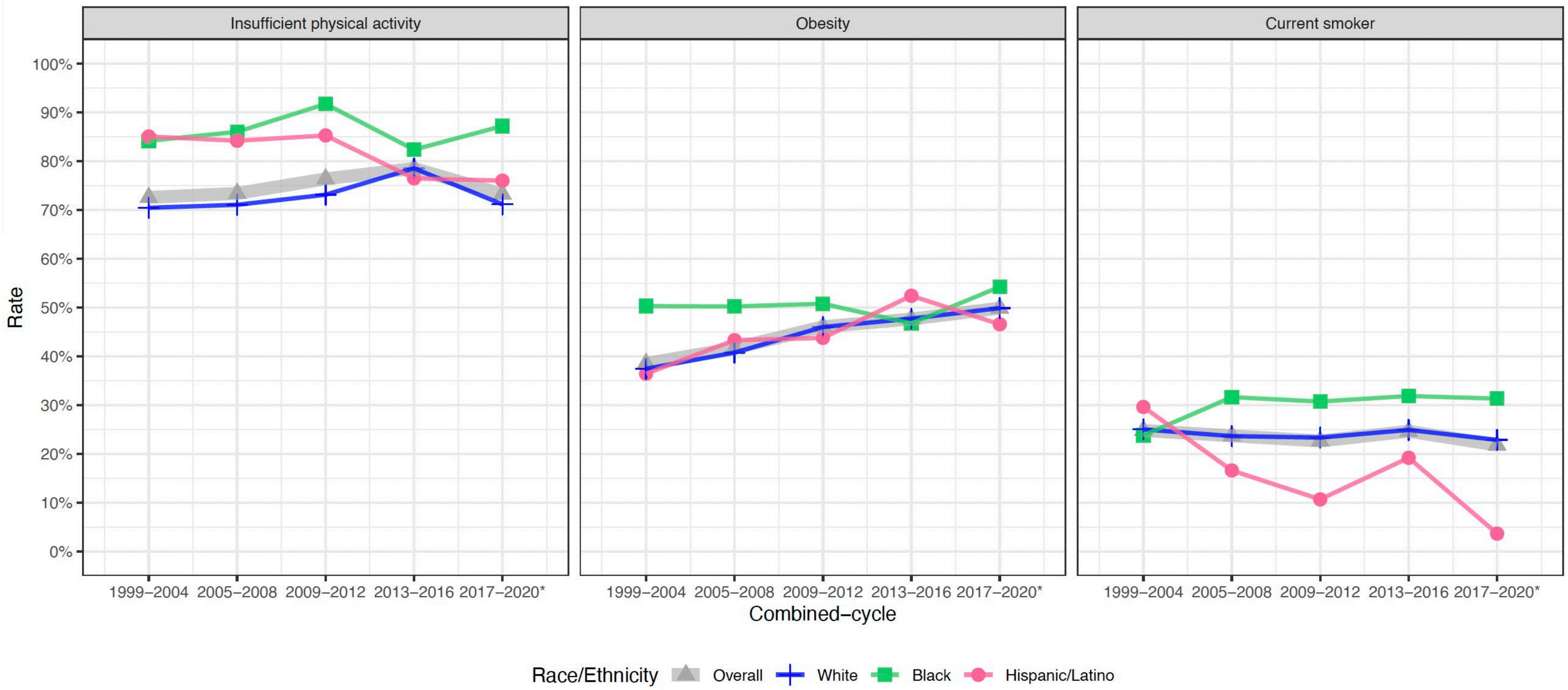

© 2023 Lu Y et al. *JAMA Network Open.*

eFigure 3. Trends in Social Determinants of Health Among Adults with Atherosclerotic Cardiovascular Disease by Race and Ethnicity, 1999-2020

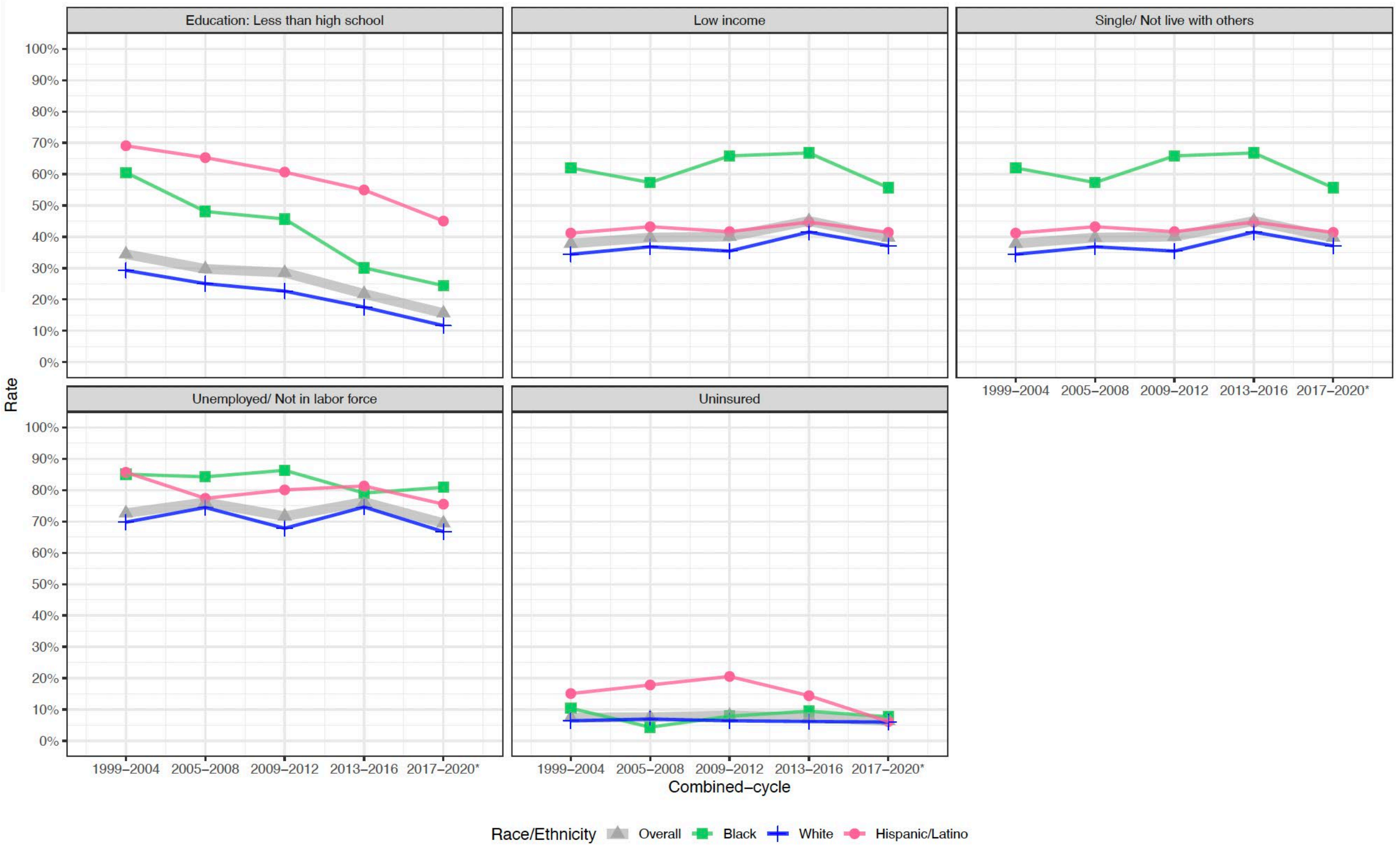

© 2023 Lu Y et al. *JAMA Network Open.*
