# Supplementary material for: National Trends in Racial and Ethnic Disparities in Use of Recommended Therapies in Adults with Atherosclerotic Cardiovascular Disease, 1999-2020
Source: JAMA Netw Open. 2023 Dec 1;6(12):e2345964. doi: 10.1001/jamanetworkopen.2023.45964 (PMC10692850; doi:10.1001/jamanetworkopen.2023.45964)
Supplement: Supplement 2. — Data Sharing Statement [file jamanetwopen-e2345964-s002.pdf]

# Data Sharing Statement

Lu. National Trends in Racial and Ethnic Disparities in Use of Recommended Therapies in Adults with Atherosclerotic Cardiovascular Disease, 1999-2020. *JAMA Netw Open*. Published December 01, 2023. doi:10.1001/jamanetworkopen.2023.45964

**Data**
**Data available:** Yes
**Data types:** Deidentified participant data
**How to access data:** The NHANES database is publicly accessible and contains deidentified data for all participants utilized in this study. This dataset can be accessed, queried, and downloaded using this link: https://wwwn.cdc.gov/nchs/nhanes/Default.aspx
**When available:** With publication

**Supporting Documents**
**Document types:** None

**Additional Information**
**Who can access the data:** Since NHANES is public, the data is always available.
**Types of analyses:** Since NHANES is public, the data is always available for any project that requires it.
**Mechanisms of data availability:** The data is always publicly accessible and can be accessed at any time.
**Any additional restrictions:** None
